# CX3CL1 release during immunogenic apoptosis is associated with enhanced anti-tumour immunity

**DOI:** 10.3389/fimmu.2024.1396349

**Published:** 2024-07-01

**Authors:** Faye Naessens, Robin Demuynck, Olga Vershinina, Iuliia Efimova, Mariia Saviuk, Greet De Smet, Tatiana A. Mishchenko, Maria V. Vedunova, Olga Krysko, Elena Catanzaro, Dmitri V. Krysko

**Affiliations:** ^1^ Cell Death Investigation and Therapy Laboratory, Anatomy and Embryology Unit, Department of Human Structure and Repair, Faculty of Medicine and Health Sciences, Ghent University, Ghent, Belgium; ^2^ Cancer Research Institute Ghent, Ghent, Belgium; ^3^ Institute of Biology and Biomedicine, National Research Lobachevsky State University of Nizhny Novgorod, Nizhny Novgorod, Russia

**Keywords:** immunogenic apoptosis, fractalkine, cytokines, immunogenic cell death, chemokines, CX3CL1

## Abstract

**Introduction:**

Immunogenic cell death (ICD) has emerged as a novel option for cancer immunotherapy. The key determinants of ICD encompass antigenicity (the presence of antigens) and adjuvanticity, which involves the release of damage-associated molecular patterns (DAMPs) and various cytokines and chemokines. CX3CL1, also known as neurotactin or fractalkine, is a chemokine involved in cellular signalling and immune cell interactions. CX3CL1 has been denoted as a “find me” signal that stimulates chemotaxis of immune cells towards dying cells, facilitating efferocytosis and antigen presentation. However, in the context of ICD, it is uncertain whether CX3CL1 is an important mediator of the effects of ICD.

**Methods:**

In this study, we investigated the intricate role of CX3CL1 in immunogenic apoptosis induced by mitoxantrone (MTX) in cancer cells. The Luminex xMAP technology was used to quantify murine cytokines, chemokines and growth factors to identify pivotal regulatory cytokines released by murine fibrosarcoma MCA205 and melanoma B16-F10 cells undergoing ICD. Moreover, a murine tumour prophylactic vaccination model was employed to analyse the effect of CX3CL1 on the activation of an adaptive immune response against MCA205 cells undergoing ICD. Furthermore, thorough analysis of the TCGA-SKCM public dataset from 98 melanoma patients revealed the role of CX3CL1 and its receptor CX3CR1 in melanoma patients.

**Results:**

Our findings demonstrate enhanced CX3CL1 release from apoptotic MCA205 and B16-F10 cells (regardless of the cell type) but not if they are undergoing ferroptosis or accidental necrosis. Moreover, the addition of recombinant CX3CL1 to non-immunogenic doses of MTX-treated, apoptotically dying cancer cells in the murine prophylactic tumour vaccination model induced a robust immunogenic response, effectively increasing the survival of the mice. Furthermore, analysis of melanoma patient data revealed enhanced survival rates in individuals exhibiting elevated levels of CD8+ T cells expressing CX3CR1.

**Conclusion:**

These data collectively underscore the importance of the release of CX3CL1 in eliciting an immunogenic response against dying cancer cells and suggest that CX3CL1 may serve as a key switch in conferring immunogenicity to apoptosis.

## 1 Introduction

Immunogenic cell death (ICD) has emerged as a paradigm-shifting concept in the field of immunology and cancer therapeutics. It combines the ability to kill cancer cells and restore the lost immunological ability to identify and interact with dying cancer cells. This leads to the stimulation of innate and adaptive immune responses, and thereby establishment of long-term immunological memory (1). ICD is an overarching term that includes cell death modalities such as apoptosis (2), necroptosis (3–5), and ferroptosis (6–8). It can be induced by specific stimuli, including oncolytic viruses (9), conventional chemotherapeutics such as mitoxantrone (MTX) (10), and physical interventions such as radiotherapy, including heavy ions (11) or X-rays radiotherapy (12), or photodynamic therapy (13). Perturbation of endoplasmic reticulum (ER) homeostasis and activation of ER stress pathways, also known as the unfolded protein response (14), together with the generation of reactive oxygen species, are essential components of nearly (15) all scenarios in which ICD occurs (16–18). For cell death to possess immunogenic properties also requires both antigenicity and adjuvanticity (1, 19, 20). Antigenicity refers to the availability of either tumour-specific antigens, tumour-associated antigens, or neo-antigens, which enable the specific recognition and killing of the tumour cells by the immune system. Adjuvanticity involves the spatio-temporal release of damage-associated molecular patterns (DAMPs) and pro-inflammatory cytokines/chemokines from the dying cancer cells. This triggers the recruitment, activation and maturation of antigen-presenting cells (APCs), such as dendritic cells, via their respective pattern recognition receptors (PRRs). Several crucial DAMPs and cytokines have been discovered, including but not limited to high-mobility group B1, surface-exposed calreticulin, extracellular secretion of adenosine triphosphate, annexin A1 and several members of the type 1 interferon family (1, 21). Upon engulfment of dying tumour cells, APCs undergo activation and maturation characterised by upregulation of major histocompatibility complex class II molecules and costimulatory markers, such as CD80 and CD86, while migrating to the tumour-draining lymph nodes to present cancer antigens to CD8^+^ T cells. The activated cytotoxic CD8^+^ T lymphocytes then relocate to the tumour site, releasing interferon gamma, promoting eradication of the neoplastic lesion, and stimulating the formation of a long-lasting immunological memory against the tumour (1, 22).

CX3C chemokine ligand 1 (CX3CL1), originally named neurotactin (23) and later fractalkine (24), is a chemokine intricately involved in cell signalling and in immune cell recruitment and activation (25). CX3CL1 can exist as a membrane-bound variant (mCX3CL1) serving as an adhesion protein for cells expressing the CX3CL1 receptor (CX3CR1), including various immune cell types such as NK cells, monocytes, dendritic cells, granulocytes, and CD3^+^ T cells (24, 26, 27). Conversely, the soluble form of CX3CL1 (sCX3CL1), which is released upon proteolytic cleavage of mCX3CL1, predominantly exerts a potent chemoattractant function (24, 25, 27–30). Importantly, CX3CL1 has been identified as a “find me” signal that attracts immune cells towards dying cells, facilitating efferocytosis (31–35). However, in the context of ICD, it remains uncertain whether CX3CL1 is an important mediator of the effect of ICD or, due to its potential pro-tumourigenic features (36–42), might act as a “keep out” signal, hindering efferocytosis and the initiation of an effective ICD immune cycle (43, 44).

In this study, we assessed the secretion of CX3CL1 from MTX-treated murine fibrosarcoma MC205 and melanoma B16-F10 cells undergoing immunogenic apoptosis (*i.e.*, apoptotic cell death exhibiting ICD characteristics (10, 45)), and explored its potential as a mediator of anti-tumour immunity during ICD. Our results demonstrate that CX3CL1 is released specifically during apoptotic cell death regardless of the cell type. Furthermore, the addition of 1 ng or 10 ng of recombinant CX3CL1 (rCX3CL1) to a non-immunogenic dose of dying/dead cancer cells for prophylactic vaccination of mice significantly increased the tumour-free survival of mice and restored immunogenicity of dying cancer cells. In addition, analysis of publicly available human database (The Cancer Genome Atlas-Skin Cutaneous Melanoma (TCGA-SKCM)) containing data from 98 melanoma patients revealed a correlation between high levels of CX3CR1 expression and higher overall survival probability. Moreover, CX3CR1 was predominantly associated with increased presence of CD8^+^ T cells, and a high level of CX3CR1 expression was correlated with increased expression of CD8^+^ T cell signature. These findings identify CX3CL1 as an effective mediator of an adaptive immune response during immunogenic apoptosis and position it as a promising therapeutic adjuvant in ICD-based treatment.

## 2 Materials and methods

### 2.1 Cell lines and cell culture

Murine fibrosarcoma MCA205 cells were cultured in Roswell Park Memorial Institute (RPMI) 1640 (Gibco, 21875–034) supplemented with heat-inactivated foetal bovine serum (FBS, Thermo Fisher Scientific, 10%, 10270–106), penicillin (100 U/mL), and streptomycin (Gibco, 100 µg/L, 15140–122). B16-F10 murine melanoma cells were cultured in Dulbecco’s Modified Eagle Medium (DMEM) (Gibco, 10938–025) supplemented with 10% FBS, 1% L-glutamine (Gibco, 25030–024), 1% sodium pyruvate (Gibco, 11360–039) and 1% penicillin/streptomycin. All cells were maintained under constant conditions of 37°C, 5% CO_2,_ and a humidified atmosphere in a cell culture incubator. The medium was changed every two days, and detaching and splitting of cells were done using trypsin-EDTA (0.05%) (Gibco, 25300–054).

### 2.2 Cell death assay by flow cytometry

Cells were stimulated with 2 µM mitoxantrone (MTX) (Sigma Aldrich, M6545) or 2.5 µM RAS-selective lethal 3 (RSL3) (Sigma Aldrich, SML2234) for 24 h. The cells were washed in Annexin-V binding buffer (10 mM HEPES, pH 7.4, 0.14 mM NaCl, and 2.5 mM CaCl_2_), followed by staining with Sytox Blue Nucleic Acid Stain (Molecular Probes, S11348, 2.5 mM), Annexin-V (AnV), and Alexa Fluor 488 conjugate (Molecular Probes, A13201). The cells were run on a Becton Dickinson (BD) LSRII flow cytometer, and the data were analysed by using FlowJo software (V.10.0.8). Flow cytometry experiments were performed at the Core Flow Cytometry (BOF/COR/2022/001) at Ghent University.

### 2.3 Multiplex analysis of cytokines

Supernatants of treated cells was analysed using the Luminex xMAP technology. The multiplexing analysis was performed using the Luminex™ 200 system (Luminex, Austin, TX, USA) by Eve Technologies Corp (Calgary, Alberta). The samples were simultaneously measured using Eve Technologies’ Mouse Cytokine Discovery Assay® (MD44). The assay was run according to the manufacturer’s protocol. Assay sensitivities of these markers range from 0.3–30.6 pg/mL. Individual analyte sensitivity values are available in the Millipore Sigma MILLIPLEX® MAP protocol.

### 2.4 Mice

The *in vivo* experiments were performed on immune-competent C57BL/6J mice (7–9 weeks old) (Janvier Labs, France). The mice were housed in specific pathogen-free conditions. All *in vivo* experiments were conducted according to the guidelines of the local Ethics Committee of Ghent University at the Core ARTH Animal Facilities at Ghent University (Belgium).

#### 2.4.1 Prophylactic tumour vaccination mice model

To confirm the role of CX3CL1 in immunogenic apoptosis, a non-immunogenic dose of MTX-treated MCA205 cells was used in the prophylactic tumour vaccination mouse model. MCA205 cancer cells were seeded at a density of 2 x 10^6^ cells per flask and induced to undergo cell death with 2 µM MTX for 24 h. After incubation overnight, 2.5 x 10^5^ MTX-treated MCA205 cells (non-immunogenic dose) or 5 x 10^5^ dying cancer cells were collected in PBS (200 µL per mouse, Gibco, 14190–144). Cell death analysis was performed using flow cytometry (See 2.2). The cells were injected subcutaneously (s.c.) in the left flank of C57BL/6J mice. After seven days, the mice were challenged with 10^5^ viable/untreated MCA205 cells in the opposite (right) flank, and tumour growth on both sides was measured with a digital calliper.

By using a non-immunogenic dose, the recovery of immunogenicity by CX3CL1 was investigated further. MCA205 cancer cells were seeded at a density of 2 x 10^6^ cells per flask and induced to undergo apoptosis with 2 µM MTX for 24 h. After incubation overnight, 2.5 x 10^5^ dying cancer cells (non-immunogenic dose) were collected and mixed with different doses (0, 1, 10 or 100 ng) of recombinant murine CX3CL1 (R&D system, 472-FF/CF) in PBS at a volume of 200 µL per mouse. Cell death analysis was performed using flow cytometry (See 2.2). The cells were s.c. injected in the left flank of C57BL/6J mice. Mice injected only with PBS or only recombinant murine CX3CL1 (1 ng, 10 ng or 100 ng) served as negative controls. Seven days post-immunisation, the mice were challenged as described above. Tumour growth was monitored with the digital calliper once every two days for up to 21 days after challenge. When a tumour became too big (> 1,500 mm^3^) or an open necrotic lesion developed, the mouse was euthanised by cervical dislocation.

#### 2.4.2 Therapeutic tumour mice model

5 x 10^5^ MCA205 cells were injected s.c. in the right flank of C57BL/6J mice. After 7 days, when the tumour had reached about 20–45 mm^3^, the mice were treated intraperitoneally (i.p.) with 100 µL of 5.2 mg/kg MTX in PBS. 12 h and 24 h after treatment, the mice were injected intratumourally with 1 or 10 ng CX3CL1 in 10 µL of PBS or, for the control mice, with PBS only. This was repeated on day 14. The efficacy of therapy was analysed by monitoring tumour growth with a digital calliper once every two days for up to 29 days after tumour cell injection. When a tumour became too big (> 1,500 mm^3^) or became an open necrotic lesion, the mouse was euthanised by cervical dislocation.

### 2.5 Public dataset

RNA-sequencing (RNA-seq) data and corresponding patient clinical information of the TCGA-SKCM project were downloaded from The Cancer Genome Atlas (https://portal.gdc.cancer.gov/). Patients with no reported vital status (alive or dead), with recurrent tumour, or with an unknown survival time were excluded. The TCGA-SKCM dataset comprises 98 patients with a primary tumour, of whom 28 have a vital status of ‘Dead’ and 70 have a vital status of ‘Alive’. For analysis, we used STAR-count files containing the number of mapped reads (counts) for each gene. The non-protein-coding genes were filtered out, leaving 19,938 genes. The expression count data were normalised by transcripts per million (TPM) and then transformed to log_2_ values.

#### 2.5.1 Survival analysis

Survival analysis of patients from the public dataset was performed in Python using the lifelines v0.27.4 package. For each specific gene, patients were divided into two groups based on median expression level (high or low). Overall survival was estimated using the Kaplan–Meier method. Log-rank test (Mantel-Cox) was used to compare the statistical differences between groups, and a p-value < 0.05 was considered statistically significant. Where survival curves intersected, a weighted log-rank test (Fleming-Harrington test) was additionally used for evaluation. Depending on the values of parameters p and q, this test can determine early (p > q) or late (p < q) differences in survival. For p = q = 0, the test reduces to the unweighted log-rank test.

#### 2.5.2 Estimation of tumour-infiltrating cells

The immunedeconv v2.0.4 (46) R package was used to analyse the abundance of immunocyte infiltration from bulk gene expression data. This package evaluates cell proportions using algorithms such as EPIC (47), TIMER (48), quanTIseq (49), MCP-counter (50) and xCell (51).

### 2.6 Statistical analysis

Statistics for the public dataset were calculated in Python using the scipy v.1.9.3 package. As the data were often not normally distributed according to the Shapiro–Wilk test, a nonparametric Mann-Whitney U test was used to evaluate the differences between two groups. P-values < 0.05 were considered statistically significant.

Statistical analysis with one-way or two-way Analysis of Variance (ANOVA) and graphs were plotted in GraphPad Prism (V.8.0.1). Kaplan-Meier survival curves showing the timelines of tumour development were analysed by log-rank Mantel-Cox test. Differences between groups were considered significant if the corresponding p-value was < 0.05.

## 3 Results

### 3.1 CX3CL1 release is associated with immunogenic apoptosis

**Figure 1:**
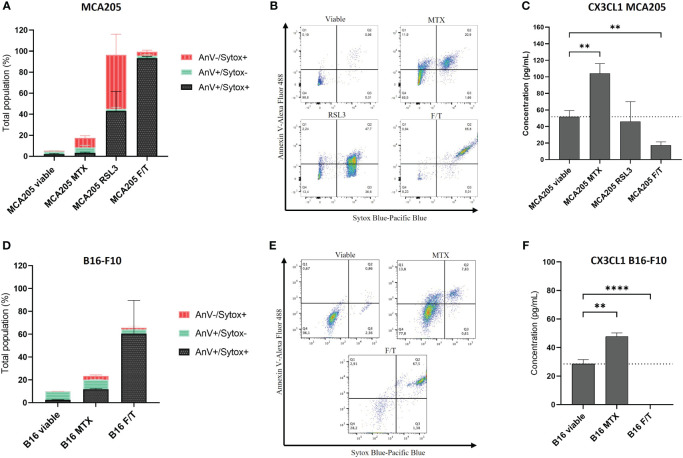
CX3CL1 release is associated with immunogenic apoptosis. **(A, D)** Cell death measured by flow cytometry of MCA205 cells **(A)** and B16-F10 cells **(D)** treated with 2 μM or 8 μM MTX (MCA205 and B16-F10 respectively), 2.5 μM RSL3, or three cycles of F/T. Quantification was done by AnV and Sytox Blue staining. The values are the means ± SEM and represent three independent experiments. **(B, E)** Representative dot plots of the cell death measurement shown in **(A, D)**. **(C, F)** The concentration (pg/mL) of CX3CL1 measured in the supernatants of dying cells using Luminex xMAP technology. The values are the means ± SEM and represent three independent experiments. Statistical significance was calculated by one-way ANOVA followed by Tukey’s multiple comparisons test: **p < 0.01, ****p < 0.0001. MTX, mitoxantrone; RSL3, RAS-lethal selective 3; F/T, freeze/thaw; AnV, Annexin-V; Sytox, Sytox Blue.

Different types of cell death modalities (*i.e.*, apoptosis, ferroptosis and accidental necrosis) were induced in both murine fibrosarcoma (MCA205) (**Figures 1A, B**) and melanoma (B16-F10) cells (**Figures 1D, E**). MCA205 and B16-F10 are commonly used cell lines in ICD studies (6, 13, 45, 52). Immunogenic apoptosis was induced with MTX for 24 h (10, 45), ferroptosis (non-immunogenic) was induced with RSL3 (6), and accidental necrosis of low immunogenicity was induced with three freeze/thaw (F/T) cycles (6, 13, 53, 54). The used cell death inducers have already been extensively described and defined (6, 10, 13, 45, 53, 54). Treatment for 24 h with RSL3 or F/T cells were used as negative controls because they do not induce the characteristics of ICD (6). Cell death rates, quantified by AnV and Sytox blue staining, were comparable with previously published data (6), *i.e.* approximately 20% for MTX (**Figures 1A, D**). Sytox Blue positivity refers to cell membrane permeabilisation and, together with positive AnV staining, detects a late phase of cell death, while single AnV positivity occurs at an early phase of cell death (6). Since B16 cells are resistant to RSL3 (55, 56), it was not used for B16. Supernatants were collected from the dying murine fibrosarcoma MCA205 and melanoma B16-F10 cells, as well as from viable cells as a control, and analysed for cytokine secretion using the Luminex xMAP technology from Eve Technologies. CX3CL1 was associated only with MTX-treatment (*i.e.*, immunogenic apoptosis) despite the limited membrane permeabilisation (**Figures 1C, F**). This release from both cancer cell types excludes the possibility of effects specific to a particular cancer cell type. During late ferroptosis in MCA205 cells, CX3CL1 levels remained unaltered compared to the viable control, whereas during accidental necrosis, the levels of CX3CL1 even diminished in both MCA205 and B16-F10 cells. These data suggest a strong association of CX3CL1 secretion with the specific induction of immunogenic apoptosis but not with the other cell death modalities.

### 3.2 CX3CL1 reverts non-immunogenic apoptosis to ICD

**Figure 2:**
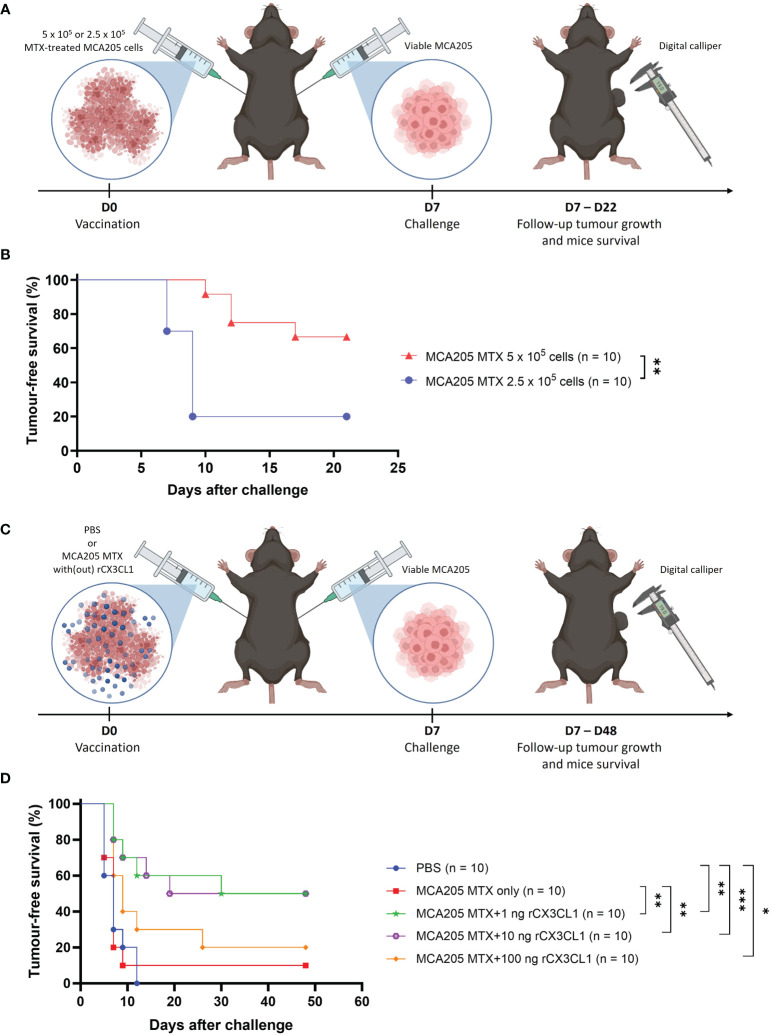
CX3CL1 reverts non-immunogenic apoptosis to ICD. **(A)** Schematic representation of the tumour prophylactic vaccination mouse model. On day 0, mice were vaccinated in the left flank with either 5 x 10^5^ or 2.5 x 10^5^ MTX-treated MCA205 cells. On day 7, the mice were challenged in the opposite flank with 10^5^ viable cancer cells of the same type and tumour growth was monitored with a digital calliper. **(B)** Kaplan-Meier curve of the progression of tumour development over time. The reduction of dose of MTX-treated MCA205 cells from 5 x 10^5^ cells to 2.5 x 10^5^ cells significantly decreased tumour-free survival from 70% to 20%. The statistical differences were calculated by a log-rank (Mantel-Cox) test. Survival curves comparison: **p < 0.01. **(C)** Schematic representation of the tumour prophylactic vaccination mouse model. On day 0, mice were vaccinated in the left flank with either PBS, 2.5 x 10^5^ MTX-treated MCA205 cells alone or 2.5 x 10^5^ MTX-treated cells in combination with different doses of recombinant CX3CL1 (1 ng, 10 ng or 100 ng). On day 7, mice were challenged in the opposite flank with 10^5^ viable cancer cells of the same type and afterwards tumour growth was followed with a digital calliper. **(D)** Kaplan-Meier curve of the progression of tumour development over time. The addition of 1 ng or 10 ng of rCX3CL1 to MTX-treated MCA205 cells significantly increased tumour-free survival from 10% (MCA205 MTX alone) to 50%. Interestingly, 100 ng of rCX3CL1 had no significant effect on tumour-free survival. The statistical differences were calculated by a log-rank (Mantel-Cox) test. Survival curves comparison: *p < 0.05, **p < 0.01, ***p < 0.001. ICD, immunogenic cell death; MTX, mitoxantrone; PBS, phosphate-buffered saline; rCX3CL1, recombinant CX3CL1.

To investigate the importance of CX3CL1 secretion in immunogenic apoptosis, the tumour prophylactic vaccination mouse model was used (**Figures 2A, C**). Mice were vaccinated with MTX-treated MCA205 cells in one flank and one week later challenged in the opposite flank with viable cancer cells of the same cancer type. For this experiment, a non-immunogenic low-dose of MTX-treated MCA205 cells was used for vaccination (**Figures 2A, B**). Vaccination of mice with 5 x 10^5^ MTX-treated MCA205 cells protected approximately 70% of the mice against tumour challenge, whereas the half-dose of 2.5 x 10^5^ cells protected only 20% of the mice (**Figure 2B**). Therefore, this so-called non-immunogenic dose of 2.5 x 10^5^ cells was used in the following experiments. Doses of 1 ng, 10 ng or 100 ng of murine rCX3CL1 were added to the non-immunogenic dose of MTX-treated cancer cells to analyse whether the addition of CX3CL1 can enhance the immunogenicity of dying cancer cells (**Figure 2C**). Indeed, addition of 1 ng or 10 ng of rCX3CL1 effectively increased tumour-free survival (p = 0.0084) in mice from 10% (non-immunogenic MCA205 MTX only) to 50%, demonstrating restored immunogenicity (**Figure 2D**). Interestingly, the addition of a dose of 100 ng rCX3CL1 to the MTX-treated cancer cells did not increase tumour-free survival and vaccination with rCX3CL1 alone (*i.e.*, without MTX-treated cancer cells) did not exhibit any effect (**Supplementary Figure S1**). These data indicate the importance of CX3CL1 secretion in establishing an effective immune response during immunogenic apoptosis, although this occurs only for an appropriate dose. Of note, the therapeutic effect of CX3CL1 during MTX treatment in MCA205 tumour-bearing mice decreased the tumour size but this decrease was not statistically significant (**Supplementary Figure S2**).

### 3.3 CX3CR1 is associated with increased CD8+ T cells and increased patient survival

**Figure 3:**
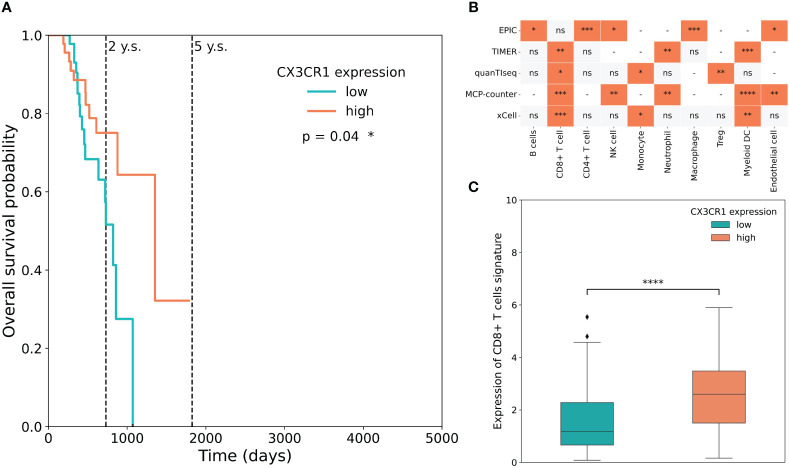
CX3CR1 is associated with increased CD8^+^ T cells and increased patient survival probability. **(A)** The association between CX3CR1 expression and overall survival (OS) in the TCGA-SKCM dataset. OS curves were generated by setting median expression as cut-off. **(B)** Statistical analysis of cell infiltration stratified based on CX3CR1 expression in the TCGA-SKCM dataset. Cellular deconvolution was performed by five algorithms (EPIC, TIMER, quanTIseq, MCP-counter and xCell). The orange square means that the abundance of cells is significantly greater in patients with a high expression level of the receptor than in those with a low expression level. A grey square means that cell proportion does not differ statistically between groups, ns – not significant. A dash means that the method does not determine the proportions of the corresponding cells. Mann-Whitney U test, *p < 0.05, **p < 0.01, ***p < 0.001, ****p < 0.0001. **(C)** Boxplots of CD8+ T-cell signature expression stratified according to CX3CR1 expression in the TCGA-SKCM dataset. High (coral) and low (cyan) expression level groups were generated by setting median expression as cut-off. Mann-Whitney U test, *p < 0.05, **p < 0.01, ***p < 0.001, ****p < 0.0001, ns, not significant.

To understand the relevance of CX3CL1 in human patients, a cohort of 98 SKCM patients was thoroughly screened utilising the publicly available TCGA dataset. We found that the presence of the receptor of CX3CL1, CX3CR1, was associated with a significantly higher five-year survival of melanoma patients (log-rank Mantel-Cox test, p = 0.04) (**Figure 3A**). Additionally, a late-weighted Fleming-Harrington test also showed a significant difference in survival (p=4.85e-03). Moreover, CX3CR1 was mainly associated with CD8^+^ cytotoxic T cells (**Figure 3B**). Finally, a high expression level of CX3CR1 in melanoma patients was correlated with increased abundance of cytotoxic CD8^+^ T-cells (**Figure 3C**). Taken together, the increase of CX3CL1 results in mobilisation and recruitment of CX3CR1-positive cells. A heightened abundance of CX3CR1 in melanoma patients, particularly associated with increased presence of CD8^+^ T cells, was correlated with increased overall survival probability among the individuals with melanoma.

## 4 Discussion

We examined the secretion of CX3CL1 from MTX-treated cancer cells undergoing immunogenic apoptosis and its potential role as a mediator of anti-tumour immunity during ICD. In our study, CX3CL1 was released exclusively during apoptotic cell death induced by MTX in murine fibrosarcoma MCA205 and melanoma B16-F10 cells. Moreover, in prophylactic vaccination of mice, the addition of 1 ng or 10 ng of rCX3CL1 to non-immunogenic doses of dying/dead cancer cells significantly enhanced tumour-free survival and restored immunogenicity of the dying cancer cells. Furthermore, analysis of the TCGA-SKCM database of data from 98 melanoma patients revealed a significant correlation between increased CX3CR1 expression and the patients’ improved overall survival probability. In addition, CX3CR1 was predominantly associated with increased abundance of CD8^+^ T cells, with high CX3CR1 expression levels correlating with increased CD8^+^ T cell signature.

Cytokines and chemokines are recognised as crucial regulators in cancer development, cell dynamics within the tumour microenvironment, and intercellular signalling processes. These molecular mediators are also an integral part of ICD, acting as signals for cellular recruitment (“find me” and “eat me” signals), as well as for immune evasion (“keep out” and “don’t eat me” signals) (1). The concept of ICD has gained significant attention as a novel immunotherapeutic strategy and is characterised by the release of DAMPs and pro-inflammatory cytokines/chemokines that can be detected by APCs via their corresponding PRRs (21). This drives the recruitment of APCs to the tumour site, facilitates recognition, engulfment, and subsequent processing and presentation of tumour antigens by APCs, and provides guidance for cytotoxic lymphocytes. CX3CL1 has been described as an important chemokine that can be implicated in the context of cancer, although controversies persist regarding the properties and activities of this chemokine due to its pro- and anti-cancer characteristics (25). But what is the role of CX3CL1 during ICD, in particular during immunogenic apoptosis?

In our experimental findings, we demonstrated that CX3CL1 was only detected in the supernatants of MTX-treated immunogenic apoptotic fibrosarcoma MCA205 and melanoma B16-F10 cells, and to a significantly lesser extent in the supernatants of viable cancer cells, RSL3-treated non-immunogenic late ferroptotic (6) cancer cells and non-immunogenic F/T accidentally necrotic cancer cells (**Figures 1C, F**). This indicates the exclusivity of CX3CL1 release during immunogenic apoptosis, suggesting its potential role in mediating immunogenicity of cancer cells undergoing apoptotic ICD. The receptor for CX3CL1, CX3CR1, is expressed on various immune cells, including NK cells, monocytes, dendritic cells, granulocytes, and CD3^+^ T cells, stimulating their adhesion, retention, and transendothelial migration to sites characterised by strong inflammatory reactions (24, 57). It has been shown that sCX3CL1 also serves as a potent chemoattractant for these CX3CR1-expressing immune cells, enabling their chemotaxis towards the cancer niche and activation of their anti-cancer functions (24, 30, 58–61). Moreover, CX3CL1 expression is crucial for dendritic cell migration, maturation, and adhesion to T cells (58), while the presence of CX3CL1 on mature dendritic cells also activates resting NK cells (62). Hence, the release of CX3CL1 by the MTX-induced dying cancer cells (**Figures 1C, F**) may establish a gradient directed towards the tumour, presumably augmenting CX3CR1^+^ immune cell migration along the gradient and activation of an anti-tumour immune cycle. Nevertheless, it is important to note that the upregulation of CX3CR1 expression may be associated with increased expression of this protein on the tumour cells, which may result in metastasis when the cancer cells enter the bloodstream and bind CX3CR1 on endothelial cells (25). However, due to the elevated concentration of CX3CL1, cancer cells expressing CX3CR1 might also be retained within the tumour and become the target of immune cells.

Furthermore, we demonstrated that the addition of CX3CL1 to a non-immunogenic dose of MTX-treated dying cancer cells is sufficient to elicit an immunogenic response in a tumour prophylactic mouse model, thereby revealing CX3CL1 as a power switch of immunogenic apoptosis induced by MTX (**Figure 2D**). Since it has been shown that CX3CL1 enhances efferocytosis of apoptotic thymocytes (34), it is conceivable that the presence of CX3CL1 in the prophylactic vaccine may lead to increased recruitment of phagocytes and clearance of dying cancer cells, consequently culminating in an overall improved anti-tumour immunity. However, the presence of CX3CL1 can also be associated with tolerogenic apoptosis (63). This underscores the nuanced and context-dependent role of CX3CL1 during cell death, where different factors intricately interact to determine the ultimate outcome. During immunogenic apoptosis, the secretion of other DAMPs fosters the anti-tumour effect, whereas during tolerogenic apoptosis DAMPs release is minimal, thus constraining the activation of an adaptive immune response. Therefore, it will be interesting to determine the differences in CX3CL1 secretion between tolerogenic apoptosis and immunogenic apoptosis, as the quantity of CX3CL1 secretion might be the key to shifting tolerogenic cell death towards an immunogenic form. In addition, it is important to note that prophylactic vaccination of mice with CX3CL1 alone (*i.e.*, without any MTX-treated cancer cells) had no effect on the tumour-free survival of the mice (**Supplementary Figure S1**), logically due to the absence of any antigens (antigenicity) or other DAMPs (adjuvanticity) during vaccination.

Remarkably, our findings from the tumour prophylactic vaccination experiments (**Figure 2D**) demonstrate that only lower doses (1 ng or 10 ng) of CX3CL1 exhibit an enhanced anti-tumour effect of ICD, whereas use of a high dose (100 ng) leads to almost complete absence of the anti-tumour effect (**Figure 2D**). This observation might indicate that the inherently inflammatory nature of CX3CL1 could contribute to hyperinflammation at higher doses, potentially promoting a pro-tumourigenic environment. Moreover, lack of sufficient DAMPs (due to a non-immunogenic dose of MTX-treated cancer cells) and excessive influx of immune cells (30, 58, 59) (due to a high CX3CL1 dose) might diminish activation of infiltrating immune cells, leading to an immunosuppressive phenotype and thus loss of the anti-tumour effect in the presence of a high dose of CX3CL1. Although gene therapy involving the transfer of CX3CL1 to cancer cells was demonstrated to induce a robust anti-cancer effect (64–66), we did not see a significant reduction in tumour size following co-treatment with CX3CL1 and MTX in the therapeutic mouse model (**Supplementary Figure S2**). It is conceivable that the supplementary CX3CL1 introduced alongside the already secreted CX3CL1 by the MTX-treated tumour cells resulted in an excessively high concentration of CX3CL1, which ceases to elicit an additive effect.

Of interest, analysis of RNA-seq data and corresponding patient clinical information of the TCGA-SKCM of 98 melanoma patients demonstrated a positive correlation between increased CX3CR1 levels and increased overall survival probability in the melanoma patients, along with a discernible CD8^+^ T cell signature (**Figure 3**). CX3CR1 has been reported to be associated with CD8^+^ T cells that respond to PD1 therapy while resisting cell death during chemotherapy (67). Moreover, CX3CR1-deficient mice injected with melanoma cells had increased tumour burden, cachexia, and defective anti-tumour responses (68). It has also been shown that an increase in CX3CL1 expression in the tumour is linked to improved prognosis of many cancer patients with breast carcinoma (69), colorectal cancer (70, 71) or lung adenocarcinoma (72), among other cancers (70–75).

In summary, our study indicates that CX3CL1 serves as a potent mediator of immunogenicity during immunogenic apoptosis induced by MTX. CX3CL1 is released by immunogenic apoptotic cancer cells regardless of the cancer cell type. Moreover, the addition of CX3CL1 to non-immunogenic doses of MTX-treated dying cancer cells in mouse prophylactic tumour vaccination models resulted in the activation of an adaptive immune response and effectively lengthened survival. In addition, an increase in CX3CR1 expression was correlated with increased overall survival probability of melanoma patients and increased CD8^+^ T cell signature. Our data provide a rationale for exploiting CX3CL1 as a future adjuvant to render therapy-induced cell death immunogenic. The addition of CX3CL1 to other treatments could affect their immunogenicity, unleashing their full immunogenic potential during cell death in anti-cancer therapy. However, to achieve an overall favourable immunogenic outcome, accurate dosing of CX3CL1 might be of paramount importance.

## Data availability statement

The original contributions presented in the study are included in the article/**Supplementary Material**, further inquiries can be directed to the corresponding author/s.

## Ethics statement

Ethical approval was not required for the study involving humans in accordance with the local legislation and institutional requirements. Written informed consent to participate in this study was not required from the participants or the participants’ legal guardians/next of kin in accordance with the national legislation and the institutional requirements. The animal study was approved by Ethics Committee of Ghent University. The study was conducted in accordance with the local legislation and institutional requirements.

## Author contributions

FN: Data curation, Formal analysis, Investigation, Methodology, Validation, Writing – original draft, Writing – review & editing. RD: Data curation, Formal analysis, Investigation, Methodology, Writing – original draft, Writing – review & editing. OV: Formal analysis, Software, Writing – review & editing. IE: Writing – review & editing. MS: Writing – review & editing. GD: Data curation, Writing – review & editing. TM: Writing – review & editing. MV: Writing – review & editing. OK: Writing – review & editing. EC: Writing – review & editing. DK: Funding acquisition, Project administration, Resources, Supervision, Writing – review & editing.
