# Supplementary material for: CX3CL1 release during immunogenic apoptosis is associated with enhanced anti-tumour immunity
Source: Front Immunol. 2024 Jul 1;15:1396349. doi: 10.3389/fimmu.2024.1396349 (PMC11246865; doi:10.3389/fimmu.2024.1396349)
Supplement: Supplementary file 1 [file DataSheet_1.docx]

Supplementary Material

# Supplementary Figures and Tables

## Supplementary Figures

**Supplementary Figure S1.** ***No effect of rCX3CL1 alone in prophylactic tumour vaccination model.*** *On day 0, mice were vaccinated in the left flank with either 1 ng, 10 ng or 100 ng of rCX3CL1 alone or in combination with 2.5 x 10^5^ MTX-treated MCA205 cells. On day 7, the mice were challenged in the opposite flank with 10^5^ viable cancer cells of the same type and tumour growth was monitored with a digital calliper. The figure shows the Kaplan-Meier curve of the progression of tumour development over time. Vaccinating mice with 1 ng, 10 ng or 100 ng of CX3CL1 alone had no improved effect on the tumour-free survival of the mice. The statistical differences were calculated by a log-rank (Mantel-Cox) test. Survival curves comparison: *p < 0.05, **p < 0.01. ICD, immunogenic cell death; MTX, mitoxantrone; PBS, phosphate-buffered saline; rCX3CL1, recombinant CX3CL1.*

**
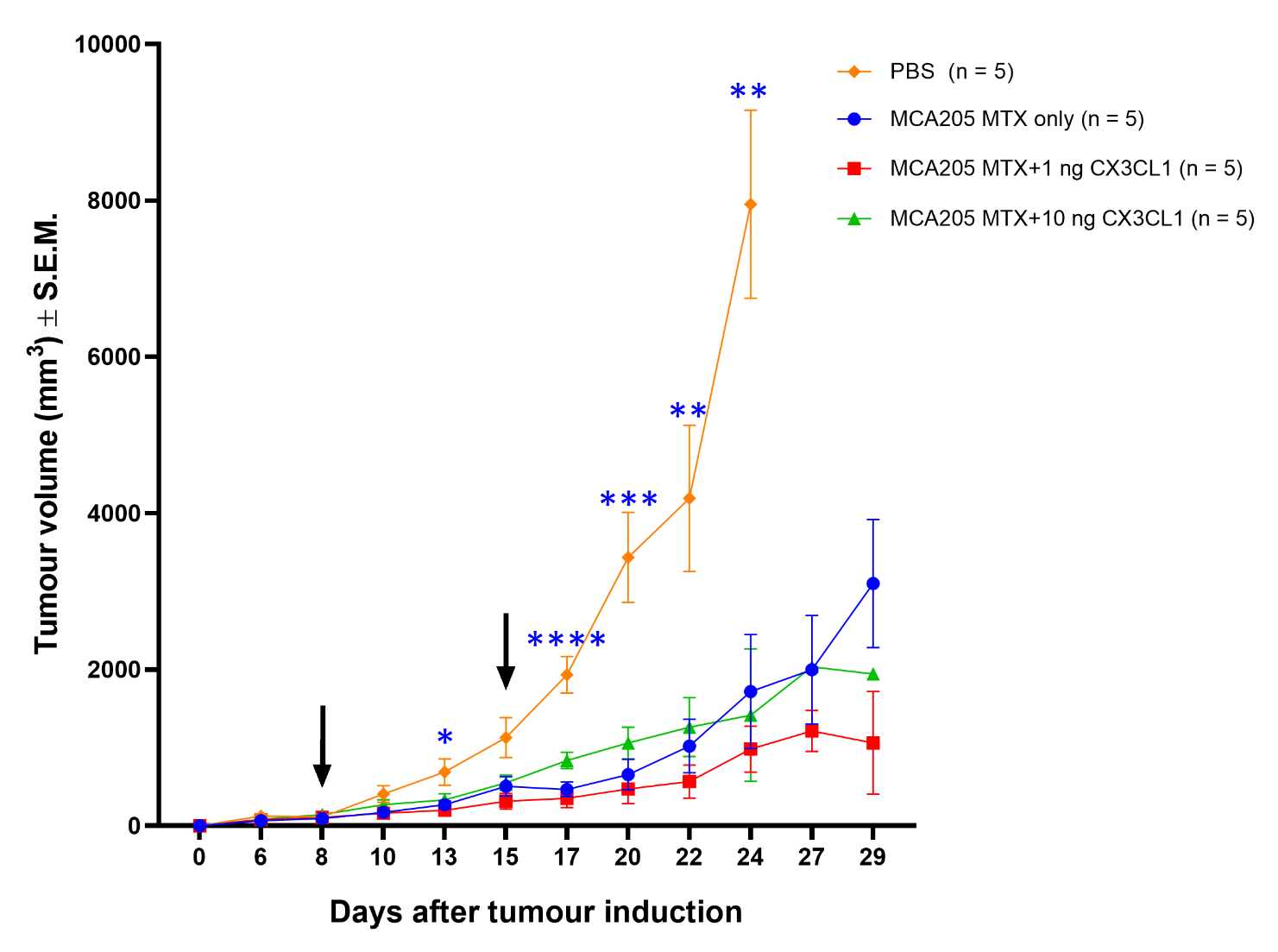
**

**Supplementary Figure S2.** ***No additive effect of CX3CL1 and MTX treatment in therapeutic regimen in mouse tumour model.*** *Tumour bearing C57BL/6J mice were i.p. treated with 100 µL PBS or 100 µL of 5.2 mg/kg MTX dissolved in PBS. 6 h and 24 h after MTX treatment, mice were intratumorally injected with 10 µL PBS, or 10 µL of PBS containing 1 ng or 10 ng of rCX3CL1. This treatment regimen was administered at day 8 and day 15 after tumour establishment. The tumour volume was followed-up until 29 days after tumour induction. Significant reduction in tumour volume was demonstrated between PBS treatment and MTX treatment. Although the addition of 1 ng or 10 ng of CX3CL1 to the MTX treatment also decreased tumour growth, no significant difference was reached between MTX treatment only and MTX treatment with the addition of 1 ng or 10 ng of rCX3CL1. Arrows indicate timepoints of treatment administration. The statistical difference between the mice treated with PBS and mice treated with MTX only were calculated by one-way ANOVA followed by Tukey’s correction for multiple comparisons, *p < 0.05, **p < 0.01, ***p < 0.001, ****p < 0.0001. MTX, mitoxantrone; i.p., intraperitoneal injection; PBS, phosphate-buffered saline; rCX3CL1, recombinant CX3CL1.*
